# Field-in-Field Technique With Intrafractionally Modulated Junction Shifts for Craniospinal Irradiation Planning With Three-Dimensional Conformal Radiation Therapy

**DOI:** 10.7759/cureus.14744

**Published:** 2021-04-28

**Authors:** Shabbir Hussain, Abdul Hafeez, Hira Nazim, Rahim Gohar, Muhammad Jawaid Mallick

**Affiliations:** 1 Oncology, Ziauddin University, Karachi, PAK; 2 Oncology, Aga Khan University Hospital, Nairobi, KEN

**Keywords:** field-in-field technique, 3d-crt, medulloblastoma, craniospinal irradiation (csi)

## Abstract

Background: Planning craniospinal irradiation (CSI) with ‘‘field-in-field’’ (FIF) homogenization technique in combination with daily, intrafractional modulation of the field junctions is needed to avoid spinal cord overdose. Photon-based techniques for CSI may result in dose inhomogeneity within the treatment volume and usually require a weekly manual shift of the field junctions to minimize the possibility of spinal cord overdose. Nowadays, FIF technique is used to feather out the dose inhomogeneity caused by multiple fields. We have started using this technique after acquiring advanced technology machines in recent years.

Methods and Materials: Sixteen patients treated with three-dimensional conformal radiation therapy (3D-CRT) for CSI were retrospectively chosen for analysis. These patients were treated during 2019-2020. Contouring of planning target volume (PTV) and organs at risk (OAR) was done, and planning was done on Varian Eclipse^TM ^Treatment Planning System (TPS) (Varian Medical Systems, Palo Alto, CA). These patients were planned with lateral craniocervical fields and posterior spinal fields using a forward-planned FIF technique. Field junctions were automatically modulated and custom-weighted for maximal homogeneity within each treatment fraction. Dose-volume histogram (DVH) was used for analysis of results. A corresponding plan without FIF technique was planned; then maximum dose at the junction was noted for each patient with both plans, and the readings were evaluated. Paired sample t-test was used to compute the p-values for the inferential statistics.

Results: Without FIF technique, the volume receiving 110% of the prescribed dose ranged from 39% to 74% (mean: 62.12%) and volume receiving 120% dose ranged from 8% to 28% (mean: 17.68%), whereas with FIF technique, the thecal sac volume receiving 110% of dose ranged from 2% to 18% (mean: 11%) and volume receiving 120% ranged from 0% to 2%. Volume receiving 100% of the dose was also calculated in both techniques; mean values of this dose range was almost similar in both groups. Later p-value was calculated, and in both dose ranges of thecal sac volume receiving 110% and 120%, the difference in values was statistically significant. Therefore, it proved that plan inhomogeneity improved with FIF technique. This technique provided consistent dose delivery during each fraction of treatment across the junctions. The maximum doses calculated at the junction were higher in the CSI plans without FIF compared to those with FIF technique.

Conclusion: This study concludes that better dose homogeneity is achieved with FIF technique as compared to non-FIF technique, and the difference in values was statistically significant.

## Introduction

Medulloblastoma is the most common malignant neoplasm of the central nervous system in children, constituting approximately 20% of all pediatric brain tumors [1]. Recently tremendous advances in technology and biology have been witnessed, resulting in an improved outcome for these children owing to refinements in micro-neurosurgery, more effective chemotherapy regimens, and modern radiotherapy techniques [2]. The current standard of care for these patients consists of maximal safe resection followed by radiation and chemotherapy, yielding a five-year survival rate of >80% for average-risk medulloblastoma and >50% for high-risk disease [3].

Radiotherapy for medulloblastoma and other central nervous system (CNS) neoplasms that are prone to cerebrospinal fluid dissemination like ependymoma, intracranial germinoma (ICG), and primitive neuroectodermal tumor (PNET) entails irradiation of the entire neuraxis, i.e., craniospinal irradiation (CSI) with a homogeneous dose. Neuraxis includes those areas drained by cerebrospinal fluid, i.e., the brain and the spinal cord up to the level of the thecal sac. Planning CSI still remains one of the most technically challenging processes in radiotherapy planning and delivery because of the need to irradiate a very large and complex-shaped target volume uniformly [1].

Generally in planning craniospinal irradiation, two laterally opposed cranial fields are matched to a posterior spine field with the potential for dose inhomogeneity at the junctions. Both the fields are matched either by gap junction method or by rotating couch and collimator ("exact-match" technique). Elder children and adults frequently require two posterior spine fields, which lead to additional junctions and planning complexity. The classical method of field edge matching is being followed in most of the radiation centers while performing CSI. In this traditional method, the junction between cranium and spine field as well as between spine fields shall be shifted twice with an extension of 0.5-1 cm upon delivering every nine Gray (Gy) or five fractions. This shifting of the junction is called as feathering technique by which the inferior margin of the cranial field is shortened and the superior and inferior portions of spine field edges are extended.

These techniques for craniospinal irradiation (CSI) may result in dose inhomogeneity within the treatment volume. A new planning methodology was developed in Varian Eclipse^TM^ Treatment Planning System (TPS) (Varian Medical Systems, Palo Alto, CA) recently worldwide. The aim of this planning is to introduce a simple method of field edge matching, thereby easy to achieve the homogeneous dose distribution in the entire PTV for CSI and to reduce the overall treatment duration. Plans were evaluated using dose-volume histogram (DVH) and dose color wash 3D dose distribution. This is called field-in-field (FIF) technique. To deploy FIF, multiple lower weighted reduction fields are created on the basis of the primary field. The reduction fields contain blocked segments strategically placed to reduce the highest isodose areas and to force greater homogeneity and conformity to the target volume.

This technique is common in other parts of the globe but has not been in practice in our country commonly, but recently we at our institute have begun planning CSI using a multisegmented, intensity-modulated ‘‘field-in-field’’ (FIF) and automated ‘‘junction-shift’’ techniques. This study will describe the cases we have treated using this method and will guide the readers about this better planning technique for CSI.

This article was previously posted to the Research Square preprint server on 31 March, 2021 (https://www.researchsquare.com/article/rs-371470/v1).

## Materials and methods

As this is a planning study only and there is no direct contact with patients, a waiver was obtained from the ethical review board of the primary institution Ziauddin University Karachi, Pakistan. Sixteen patients (two adults and 14 children) treated with 3D-CRT for craniospinal irradiation were retrospectively chosen for this analysis. Fourteen of these patients were diagnosed cases of medulloblastoma, while one patient had an atypical teratoid rhabdoid tumor (ATRT), and another had a germ cell tumor of brain. These patients were planned and treated during 2019-2020.

Computed tomography simulation

Planning computed tomography (CT) scans of these patients were done with intravenous contrast on the SOMATOM Definition AS Open CT Simulator (Siemens, Munich, Germany). Patients, which included mostly children, were positioned supine with a hyperextended neck rest so as to avoid divergence of the posteroanterior (PA) field through the oral cavity. A thermoplastic facemask with an extended neckline was used for head immobilization, and a shoulder retractor was used for retracting the shoulder as low as possible to extend the lateral craniocervical fields. Furthermore, a leg-separator was used to immobilize the lower body. Some of the younger children needed anesthesia for CT planning, and an anesthetist was called for it. Planning CT images of each patient were acquired from the vertex to the coccyx. After performing the CT simulation with all its requirements, DICOM (Digital Imaging and Communications in Medicine) images with 3-mm thickness were transferred to the Varian Eclipse^TM^ Treatment Planning System, version 13.0.

Image registration, contouring, and approval

Previous (preoperative) diagnostic MRI brain and spine images of the patients were retrieved from the local Picture Archiving and Communication System (PACS) of the institution. These images were registered with the CT simulation images using the registration option of the TPS. Target volume delineation was performed in accordance with internationally accepted guidelines [4]. Gross tumor volume (GTV) in this case would only be any postoperative residual area, and it will be of help only in the second phase of radiation, i.e., posterior fossa boost. Contouring of the clinical target volume (CTV) includes the brain, meninges, and spinal cord up to the thecal sac, which ends at S2 vertebrae and includes a region of the optic nerve, exiting nerve origins in the base of the skull, and posterior dorsal nerve roots, which are the areas of cerebrospinal fluid (CSF) drainage. These volumes were drawn with the help of the previous MRI images and consultation with the radiologist of the institution. The planning target volume (PTV) was generated by growing a uniform volumetric margin of 5 mm in all directions over the corresponding CTVs. OARs outlined included the eyes, heart, lungs, esophagus, liver, and kidneys. These contours were first prepared by the postgraduate residents of the radiation oncology program, which were then reviewed by the attending radiation oncologist. Later, all the volumes were peer-reviewed finally by the whole radiation oncology team including the planners, i.e., the dosimetrist and medical physicist for any final change in contours.

3D-CRT planning with FIF feathering technique at the junction

Planning was done on Varian Eclipse^TM^ Treatment Planning System, version 13.0. All of these patients were planned with lateral craniocervical fields and posterior spinal fields using a forward-planned FIF technique. Another plan was made using the non-FIF technique. The craniocervical fields used were parallel opposed, asymmetric jaws were used to block the half beam, and isocenter was kept at the cervical spine. In addition, multileaf collimators (MLC) were used to confirm the field to the PTV and to shield the critical organs. Half beam was blocked from the isocenter to provide a nondivergent junction with the posterior spine field with couch and collimator rotations.

Matching the upper border of the spine field to the lower border of the cranial field requires strict attention to achieve accuracy; overlapping of the spinal field to the cranium (i.e., overdosing) may lead to catastrophic outcomes for the patient. Uncertainty of dose distribution in this sensitive region is eliminated by adopting half-beam blocked fields and then eliminating the under- and over-dosage with feathering techniques.

**Figure 1:**
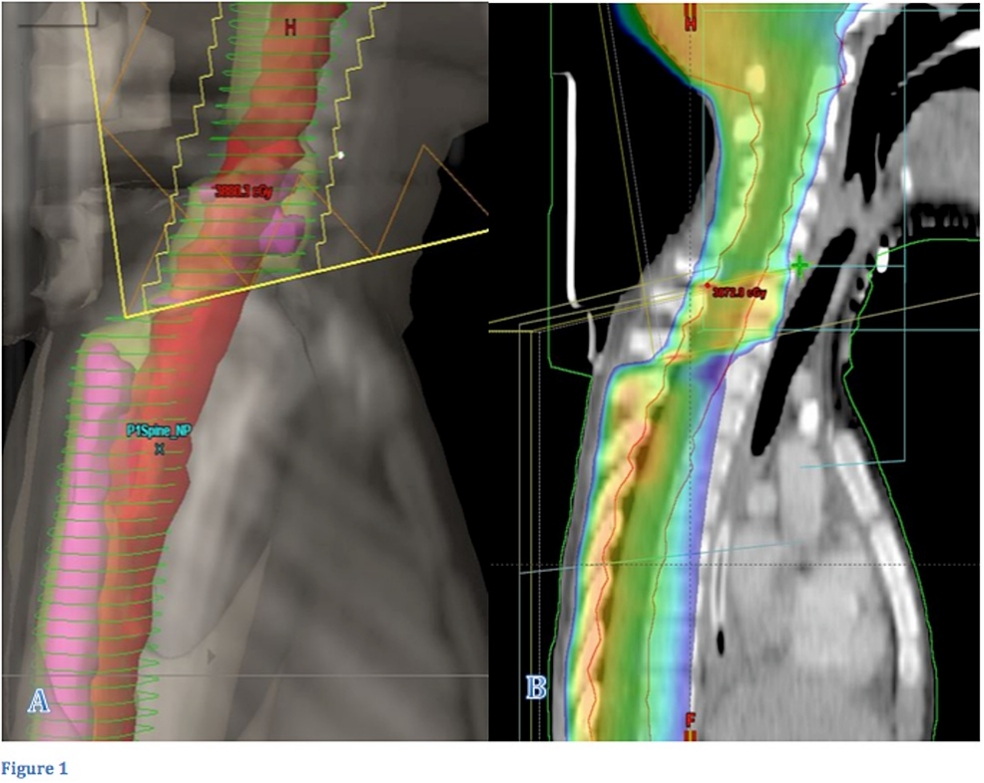
Comparison of two plans of a medulloblastoma child patient with and without field-in-field technique and modulated junctions A: Sagittal DRR of a medulloblastoma child planned without field-in-field technique. Note the junction of the craniocervical field with the PA field showing deficient coverage of the anterior area of the PTV with 95% coverage (shown in green lines) of the prescribed radiation dose and the subsequent hot spot areas showed with purple-colored dose distribution areas. B: Sagittal CT images of the same patient planned with field-in-field technique and modulated junctions, and dose coverage is showed in color wash. The junction can now be seen covered with ≥95% coverage of the prescribed dose with minimum hot spot areas. DRR, Digitally reconstructed radiograph; PA, posteroanterior; PTV, planning target volume.

Field junctions were modulated and custom-weighted for maximal homogeneity within each treatment fraction. At the craniospinal match in the low neck, multileaf collimator leaf adjustments were used at the junction field edges; and these were shifted by successive 1-cm increments during each treatment session. Three approximately weighted control points were used, with two successive 1-cm shifts in each fraction of treatment. More control points were used as needed so as to homogenize the dose distribution across the junction. To assess the predicted coverage in the plan evaluation, thecal sac volumes were contoured with 1-cm margins around the superior and inferior borders of the modulated segments. Plans were optimized, and the assigned physicist/dosimetrist evaluated the plan so that it can be practically reproduced on the treatment machine (Figure 1).

Plan evaluation and treatment delivery

DVH was used for analysis of results. Maximum dose to spinal cord and lens was evaluated to be within tolerance and was observed that the maximum dose to the spinal cord is less than 44 Gy and the maximum dose to the lens is less than 5 Gy. Treatment plans were reviewed by the radiation oncologist. As per department protocol, after the primary attending physician, plans were put on a weekly planning review meeting and were approved by the whole oncology team including the oncologists and medical physicists. Portal dosimetry was also done to assess the actual dose delivered at the modulated junctions. Treatment was then executed in the Varian Trilogy^TM^ machine (Varian Medical Systems, Palo Alto, CA).

**Figure 2:**
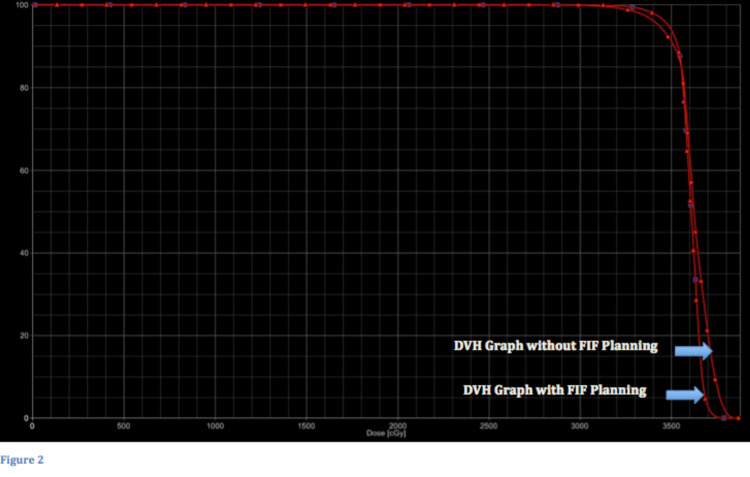
Comparison of DVH of the same patient planned with and without FIF technique The graph shows two curves, one without FIF technique and modulated junction of which the curves show deficient 95% coverage and along with that more hot spot. In contrast, the second curve with the FIF technique shows good 95% coverage with lesser hot areas. DVH, Dose-volume histogram; FIF, field-in-field.

For this study, a corresponding plan without FIF technique was planned, and maximum dose (in terms of volume of the PTV covering ≥100%, ≥110%, and ≥120%) at the junction was noted for each patient; then mean was calculated by averaging all values and dividing with the total reading in each volume with both plans, and the readings were evaluated. Statistical analysis was done to check the significance of the difference of values. Paired sample t-test was used to compute the p-values for inferential statistics (Figure 2).

## Results

Improvement in dose conformality and reduced inhomogeneity

FIF technique improved conformality of dose to the thecal sac; intrafractional modulation of doses at the field junction also helped to reduce the maximum dose on the thecal sac. Without FIF or modulated junctions, the volume receiving 110% of the prescribed dose ranged from 39% to 74% (mean: 62.12%) and volume receiving 120% dose ranged from 8% to 28% (mean: 17.68%), whereas with FIF and modulated junctions, the thecal sac volume receiving 110% of dose ranged from 2% to 18% (mean: 11%) and volume receiving 120% ranged from 0% to 2% (Table 1).

**Table 1:** Dose to thecal sac evaluated with and without FIF technique and modulated junction shifts This table shows the dose at the thecal sac with and without FIF technique, and it proves that with field-in-field technique and modulated junctions, doses ≥110% and ≥120% are recorded very much less to the thecal sac as compared to non-FIF technique. FIF, Field-in-field.

S. No.	Patient Diagnosis	Volume of Thecal Sac at ≥100%, ≥110%, and ≥120% of the Prescribed Dose					
		Without FIF Technique			With FIF Technique		
		≥100%	≥110%	≥120%	≥100%	≥110%	≥120%
1.	Medulloblastoma	99	71	16	98	18	0
2.	Medulloblastoma	99	68	21	99	15	0
3.	Medulloblastoma	98	72	6	99	15	0
4.	Atypical teratoid rhabdoid tumor	99	74	15	97	10	0
5.	Medulloblastoma	97	73	25	99	11	0
6.	Medulloblastoma	99	55	10	97	6	1
7.	Medulloblastoma	98	71	20	99	10	0
8.	Germ cell tumor brain (germinoma)	99	69	26	97	15	2
9.	Medulloblastoma	99	68	28	99	5	0
10.	Medulloblastoma	98	67	18	98	2	0
11.	Medulloblastoma	97	54	27	99	11	0
12.	Medulloblastoma	98	40	19	98	9	0
13.	Medulloblastoma	99	65	15	99	12	1
14.	Medulloblastoma	97	39	8	97	14	0
15.	Medulloblastoma	98	45	12	96	13	0
16.	Medulloblastoma	99	63	17	99	10	1

Further on statistical analysis, it was recorded that the difference of volumes of thecal sac covering 110% and 120% of the radiation dose with FIF technique as compared to non-FIF technique was statistically significant as calculated with paired sample t-test. The p-value was <0.0001 in both volumes, whereas the volume of thecal sac covering 100% of the prescribed radiation dose had no significant difference in both groups (Table 2).

**Table 2:** Comparison of means and p-value calculation with and without FIF technique and modulated junction shifts FIF, Field-in-field.

Volume of Thecal Sac at ≥100%, ≥110%, and ≥120% of the Prescribed Dose	Mean Scores	P-Value
≥100% (without FIF technique)	98.31	0.580
≥100% (with FIF technique)	98.12	
≥110% (without FIF technique)	62.12	<0.0001
≥110% (with FIF technique)	11.00	
≥120% (without FIF technique)	17.69	<0.0001
≥120% (with FIF technique)	0.31	

Practical implementation of modulated junction shifts

First, a single consistent cranial setup point was used throughout the entire radiation course due to which treatment was shortened and simplified, and the delivery process was made less prone to manual errors in the setup of the junctions. Second, portal dosimetry confirmed the safety of modulated treatment delivery with no point in the junction region in the lower spine reaching >80% of the prescription dose measured in the coronal plane or >99% measured in the axial plane. The dose was delivered as planned.

## Discussion

CSI is a mode treatment in many pediatric and some adult brain tumors. Mostly done in cases of medulloblastoma, other areas where CSI is used include anaplastic ependymoma, intracranial germinoma, PNET, and others. Medulloblastoma is the most common type of malignant brain tumor in childhood with an incidence of 18%-20% of all brain tumors [4]. As most patients in this study are cases of medulloblastoma, we need to know about it. For treatment purposes, patients with medulloblastoma are divided into two prognostic groups: children over 3-5 years of age with nonmetastatic disease and minimal residual disease (<1.5 cm^2^) post‐operatively comprise the standard‐risk group. Other patients with a subtotal resection or metastatic disease and younger patients below 3-5 years of age comprise the high‐risk group [5]. Irrespective of the risk group, CSI with or without chemotherapy is the standard of treatment for medulloblastoma [6]. In standard-risk disease (defined as gross totally resected tumor without severe anaplasia and no metastases), 23.4 Gy to the craniospinal axis plus a boost to 54 Gy to the posterior fossa, followed by adjuvant chemotherapy, is standard and has resulted in five-year survival of 80% or better [3].

Craniospinal irradiation still remains one of the most technically challenging processes in radiotherapy planning and delivery because of the need to irradiate a very large and complex-shaped target volume homogenously. With continuous improvements in long-term survival, particularly in children with average-risk medulloblastoma, there is a growing concern regarding treatment-related long-term side effects. Some of these side effects include neurocognitive decline, hearing impairment, growth retardation, endocrine dysfunction, cataract formation, cardiomyopathy, impaired fertility, and second malignancies [1]. Other than the long-term complications, PTV coverage is equally important in terms of high incidences of neuraxial recurrences, which is supported by many studies [7].

Before the advent of CT-based radiation treatment planning, CSI was done in a very old-fashioned way. Patients were set to a prone position, and two plans with different isocenters were used to treat cranium and whole spinal cord. The first plan with right and left lateral beams covered the cranium and the upper cervical vertebrae till the shoulder clearance. Taking the maximum neck area in the lateral fields gives a chance to save the oral cavity and oropharynx in getting treated with high doses of radiation. The second field covers the remainder of the spine with PA projection; the junction of both plans is kept at the neck level where the cold spot toward the posterior and hot spot toward the anterior side are created due to the divergence of the junction fields. For adult patients or tall children, the posterior spinal field cannot cover the whole remaining spine in a single field due to mechanical limitations of 40 cms, and there were two PA fields used; junction of these fields was to be matched and feathered as well. Lead shielding is applied to the craniocervical field, blocking the orbits, oral cavity, and oropharynx with keeping margins to the target spinal cord area. For the feathering gap, junction shifting is done, i.e., for every 9 Gy or five fractions, extend the cranial field superiorly by 1 cm, shift the upper spine field superiorly by 1 cm, and extend the lower spine field by 1 cm.

In the new era of radiotherapy, 2D treatments for CSI have now been minimized even in the developing nations, and it has now been replaced with 3D-CRT at least followed by intensity-modulated radiotherapy/volumetric modulated arc radiotherapy (IMRT/VMAT) where facilities are available. 3D-CRT technique for CSI has been described in detail in the section above, but the new variation in 3D-CRT that is now being used for feathering in CSI is the FIF technique along with intrafractional modulation. In this variation, multiple subfields are created of a single field with different weightages, and in a single fraction of radiotherapy, doses are modulated as these subfields ultimately create feathered doses at the junctions; in this way, setup variation at the junctions was minimized because each junction was self-feathering within each fraction [8]. This technique is also called forward-planned IMRT. This variation of 3D-CRT technique of CSI increases dose homogeneity in the target volume while decreasing the absorbed dose in the irradiated tissues outside the targeted tissue [9]. Furthermore, pediatric patients do not usually require the addition of many reduction fields because of the relatively uniform depth of the spinal column; but in adults, several fields may be required, which can be weighted as much as 10%-12% per field in the lower spine. As a forward-planned, step-and-shoot process, the use of FIF does not add substantially to the time and effort required for planning and treatment delivery [8].

## Conclusions

This study concluded that the FIF technique used for CSI planning with 3D-CRT proved to be a good method to achieve a near to normal feathering at the junctions with lesser time to treatment in the machine. It is a good alternative to modern methods of radiotherapy like IMRT and VMAT.
